# Wall shear stress gradient is independently associated with middle cerebral artery aneurysm development: a case-control CFD patient-specific study based on 77 patients

**DOI:** 10.1186/s12883-021-02251-3

**Published:** 2021-07-19

**Authors:** Mikołaj Zimny, Edyta Kawlewska, Anna Hebda, Wojciech Wolański, Piotr Ładziński, Wojciech Kaspera

**Affiliations:** 1 Department of Neurosurgery, Medical University of Silesia, Regional Hospital, Sosnowiec, Poland; 2 Department of Biomechatronics, Silesian University of Technology, Zabrze, Poland; 3 Maria Sklodowska-Curie National Research Institute of Oncology, Gliwice, Poland

**Keywords:** Middle cerebral artery, Intracranial aneurysm formation, Wall shear stress, Wall shear stress gradient, Computational fluid dynamics, Haemodynamics

## Abstract

**Background:**

Previously published computational fluid dynamics (CFD) studies regarding intracranial aneurysm (IA) formation present conflicting results. Our study analysed the involvement of the combination of high wall shear stress (WSS) and a positive WSS gradient (WSSG) in IA formation.

**Methods:**

We designed a case-control study with a selection of 38 patients with an unruptured middle cerebral artery (MCA) aneurysm and 39 non-aneurysmal controls to determine the involvement of WSS, oscillatory shear index (OSI), the WSSG and its absolute value (absWSSG) in aneurysm formation based on patient-specific CFD simulations using velocity profiles obtained from transcranial colour-coded sonography.

**Results:**

Among the analysed parameters, only the WSSG had significantly higher values compared to the controls (11.05 vs − 14.76 [Pa/mm], *P* = 0.020). The WSS, absWSSG and OSI values were not significantly different between the analysed groups. Logistic regression analysis identified WSS and WSSG as significant co-predictors for MCA aneurysm formation, but only the WSSG turned out to be a significant independent prognosticator (OR: 1.009; 95% CI: 1.001–1.017; *P* = 0.025). Significantly more patients (23/38) in the case group had haemodynamic regions of high WSS combined with a positive WSSG near the bifurcation apex, while in the control group, high WSS was usually accompanied by a negative WSSG (14/39). From the analysis of the ROC curve for WSSG, the area under the curve (AUC) was 0.654, with the optimal cut-off value −0.37 Pa/mm. The largest AUC was recognised for combined WSS and WSSG (AUC = 0.671). Our data confirmed that aneurysms tend to form near the bifurcation apices in regions of high WSS values accompanied by positive WSSG.

**Conclusions:**

The development of IAs is determined by an independent effect of haemodynamic factors. High WSS impacts MCA aneurysm formation, while a positive WSSG mainly promotes this process.

## Background

Intracranial aneurysms (IAs) are defined as pathological dilations of intracranial arteries caused by a weakness within the arterial wall. They are a relatively common disease with a worldwide prevalence of approximately 3% [1]. While often asymptomatic, cerebral aneurysms may rupture, causing a life-threatening condition – subarachnoid haemorrhage (SAH). The vast majority of aneurysms are located in the anterior part of the circle of Willis (the anterior communicating artery, ACoA 18–24%; the internal carotid artery, ICA 37–42%) and in the middle cerebral artery (MCA, 27–35%) [1–3]. As IAs tend to develop near certain bifurcations and along vessel curvatures it is hypothesised that the local haemodynamic environment may play a key role in IA formation [4, 5].

Although the morphometric and demographic risk factors responsible for aneurysm growth and rupture were thoroughly tested, researched and established [6–8], the exact pathophysiology of aneurysm formation initiation, including haemodynamic factors, remains unclear. Previously published data have shown that the vascular segments mentioned above are affected by complex haemodynamic forces, such as: wall shear stress (WSS), the wall shear stress gradient (WSSG), and temporal fluctuations in WSS [4, 5, 9–16].

Histologic findings revealed that both high and low WSS values may be responsible for initiating the formation of IAs. High WSS induces wall remodelling by the migration of smooth muscle cells, the secretion of inflammatory mediators and endothelial injury [17–19]. Low WSS may lead to localised degeneration of the arterial wall by favouring disorganisation and apoptosis of the endothelium, as well as oxidative stress and additional inflammation [20–23]. Additionally, a positive WSSG downregulates expression of several genes inhibiting proliferation, promoting inflammation and increasing secretion of ADAMTS1 protease [24, 25]. The above changes may result in increased susceptibility of the arterial wall to mechanical damage caused by local blood flow.

A significant number of image-based computational fluid dynamics (CFD) studies connecting specific haemodynamic parameters with intracranial aneurysm growth and rupture have been published in recent years but they present conflicting results, correlating both high and low WSS values with aneurysm growth and rupture or denying the importance of previously analysed parameters, introducing new ones in their place [12, 13]. The above complications may arise from conducting research on relatively small sample groups of patients or using the same averaged velocity profile in every CFD simulation [14]. Therefore, against this background, we designed a case-control study with a selection of patients with an MCA aneurysm and non-aneurysmal controls to determine the involvement of WSS, the WSSG and the oscillatory shear index (OSI) in aneurysm formation based on patient-specific CFD modelling. To the best of our knowledge, this is one of the first CFD studies in which numerical simulations were carried out based on patient-specific velocity profiles obtained from a transcranial Doppler ultrasound. This study is part of a wide range of research studies aimed at confirming the results of clinical observations, experimental research on animals and numerical analyses explaining haemodynamic involvement, mainly a combination of high WSS values and positive WSSG, in aneurysm formation.

## Methods

### Data source

The inclusion criteria for the case group were as follows: male or female adults, aged 18–75 years, and diagnosed with an unruptured MCA aneurysm. Between June 2013 and June 2017, 38 patients with unruptured MCA aneurysms diagnosed using three-dimensional computed tomography angiography (3D CTA) were enrolled in the case group. The case group consisted of seven males and 31 females ranging from 37 to 75 years old, with a mean age of 56 years. All patients had saccular and unruptured aneurysm located on the MCA bifurcation. The control group consisted of 39 cases of patients with no cerebrovascular diseases, 19 males and 20 females with a mean age of 50 years (ranging from 20 to 72). Every patient in the control group had a CTA done in order to exclude a suspected intracranial aneurysm or to establish the aetiology of minor symptoms, such as headache or vertigo.

Patients suffering from other vascular malformations that could potentially affect cerebral blood flow, haemodynamically significant carotid artery stenosis or connective tissue disorders (e.g. Marfan syndrome, Loeys-Dietz syndrome and Ehlers-Danlos syndrome types II and IV) were excluded from this study. Additional exclusion criteria from the study were: age under 18 years or over 75 years, presence of multiple cerebral aneurysms (other than mirror MCA aneurysms) or other than aneurysm pathologies in the central nervous system that could have a potential effect on cerebral blood flow, severe systemic disorders, severe heart or multi-organ failures, pregnancy and family history of cerebral aneurysm or genetically determined conditions associated with an increased risk of intracranial aneurysm formation (e.g. autosomal dominant polycystic kidney disease, neurofibromatosis type I, multiple endocrine neoplasia type I, pseudoxanthoma elasticum, hereditary haemorrhagic telangiectasia).

The study’s protocol was approved by the Institutional Review Board at the Medical University of Silesia in Katowice, Poland and all procedures were carried out in accordance with the relevant guidelines and regulations. Each patient was informed about the purpose and course of the research and asked to give their informed consent to participate in the project.

### CTA and transcranial colour-coded sonography (TCCS) protocols

All CT studies were performed using a 64-row 128-slice CT system (GE Optima CT 660, GE Healthcare, USA) using the parameters previously described [26]. On average, 50 ml of ionic contrast material (Iomeron 350, Bracco Imaging Deutschland GmbH, Konstanz, Germany) was injected into the basilic vein at 4–4.5 ml/sec via a selectively positioned catheter with the aid of an automatic syringe (OptiVantage DH, Mallincrodt, St Louis, MO, USA).

**Fig. 1:**
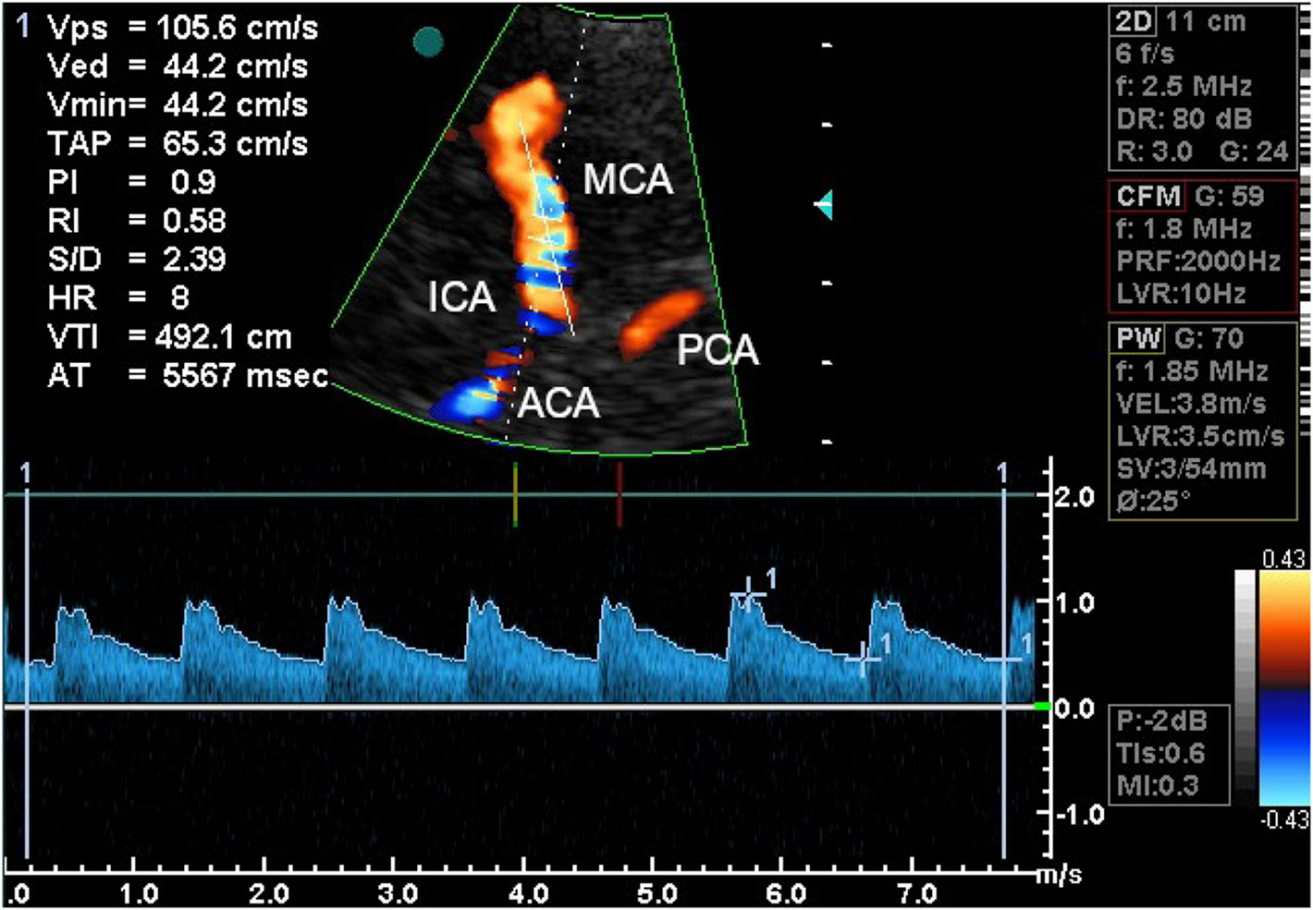
View of a colour-coded image of the middle cerebral artery (MCA) with a corresponding Doppler spectral analysis performed using a transtemporal insonation. The sample volume was placed at a depth of 54 mm in the distal portion of the MCA. ICA – the internal carotid artery; PCA – the posterior cerebral artery; A1 ACA – A1 segment of the anterior cerebral artery

All TCCS examinations were performed by the same researcher (WK) using a Vivid 3 Pro (GE Healthcare, Chicago, Illinois, USA) equipped with a multi-frequency transcranial probe (1.5–3.6 MHz) through the temporal acoustic window, in accordance with the previously described standards [27]. Anterior cerebral circulation was imaged through the temporal acoustic window with the subject in a supine position. The angle-corrected mean blood-flow velocity (Vm), peak systolic velocity (Vps) and end-diastolic velocity (Ved) were measured for both MCAs (Fig. 1). The spectral Doppler mode was used to graphically display the spectrum of flow velocities that was exported to a graphic file (i.e. BMP format file) and then used in the numerical modelling steps. The groups were analysed in terms of their applied velocity profiles and no statistical differences were found between the case and control groups (Vps: 97.4 vs 99.1 [cm/s], *P* = 0.665; Ved: 44.0 vs 43.9 [cm/s], *P* = 0.586; Vm: 66.4 vs 64.8 [cm/s], *P* = 0.554; respectively).

### Model construction

The patient-specific models were generated from CTA images using Mimics v16.0 and 3-matic v8.0 (Materialise, Leuven, Belgium). Any touching vessels and lesser branches were removed so the models only consisted of a trunk a bifurcation and the main branches arising from it. Aneurysms were manually removed, and the pre-aneurysmal geometries were recreated. Afterwards, the models were smoothed. Inlet branches were clipped at a manually selected location at the start of the artery perpendicular to its centreline to allow development of the flow. The outlets were clipped as close to the ends of the model as possible. By doing so, we eliminated model inaccuracies that might have affected the final results. Cross-sectional cuts before and after aneurysm removal were inspected to verify if the reconstructed artery was as intended.

The 3D models were pre-discretized and optimized in 3-matic software and subsequently imported as *.igs files to ANSYS Workbench (ANSYS Inc., Canonsburg, PA, USA) and computational meshes were generated using the ANSYS meshing module. The mesh was composed of tetrahedral elements with a fixed side length of 0.3 mm. To ensure an accurate definition of the velocity gradient, three inflation layers were included near the wall. The inflation growth rate was set to 1.2 and the maximum thickness was 0.15 mm. On average, computational meshes in the case group consisted of 810,000 tetrahedral prismatic elements and 740,000 in the control group.

### Numerical modelling

Navier-Stokes equations describing blood flow were solved by performing CFD simulations using an ANSYS Fluent, a finite volume-based CFD solver. Blood was considered to be an incompressible Newtonian fluid with a density of 1056 kg/m^3^ and a constant viscosity of 0.0035 Pa*s. Arterial walls were assumed to be rigid and a no-slip boundary condition was applied at them. Boundary conditions describing patient-specific inlet velocity profiles were based on the MCA blood-flow waveforms measured with TCCS and analysed by a custom-written script (see link: https://github.com/kudlacz964/tccs2ansys). The programme transcribed the BMP graphic files acquired from the TCCS into numerical files readable by the ANSYS computing environment for each patient. The Python Imaging Library used in the script allowed for highly accurate readings. An initial value of 0 was used for the pressure at both outlets.

The length of the time step was 0.001 s and the duration of the simulation was dependent on the duration of the patient’s cardiac cycle. To establish convergence in every time step, two cardiac cycles were calculated and only the data obtained from the second cycle was analysed.

### Haemodynamic parameters

The following parameters were analysed within a defined circular patch with a radius of 5 mm and a centre in the bifurcation apex, which was consistent among the subjects and encapsulated the aneurysm initiation sites in the dataset.

WSS is a tangential, frictional stress exerted on the vessel wall by the flow of the blood. During the research, the time-averaged WSS was analysed, which was calculated by averaging the WSS vector over the cardiac cycle. In this study, a high WSS was defined as time-averaged WSS values in the 98th percentile for each case.

$$ WSS=\frac{1}{T}{\int}_0^T\left|{wss}_i\right| dt $$

where *wss*_*i*_ is the instantaneous WSS vector and *T* is the duration of the cardiac cycle.

The WSSG was calculated by taking the spatial derivative of the time-averaged WSS. Apart from the WSSG, additional WSSG-derived parameters were also distinguished: absolute WSSG (absWSSG) and WSSG direction (dirWSSG). The WSSG was defined as the WSSG averaged over the high WSS area. The WSSG measurements were taken from one branch – in the case group, from the branch developing aneurysm or from the branch with the higher WSS values when aneurysm was big enough to cover both branches or the bifurcation apex. In the control group, the WSSG measurements were taken from branch with the higher WSS values.

The dirWSSG was the categorical variable: positive (dirWSSG+) and negative dirWSSG (dirWSSG-). This variable was positive when the WSSG vector direction was the same as the WSS vector originating from the same point, while for dirWSSG, it was the opposite.

$$ WSSG=\sqrt{{\left(\frac{\partial {\tau}_{w,p}}{\partial p}\right)}^2+{\left(\frac{\partial {\tau}_{w,q}}{\partial q}\right)}^2} $$

where *τ*_*w*_ is the WSS vector, the *p*-direction corresponds to the time-averaged direction of the WSS, and the *q*-direction is perpendicular to *p*.

OSI was introduced to measure changes in the direction of WSS during the cardiac cycle. Its values range from 0.0 in the case of unidirectional flow to 0.5 in regions of high and complete departure of the WSS vector from its original axis. The OSI was defined as the OSI averaged over the high WSS area.

$$ OSI=\frac{1}{2}\left(1-\frac{\left|{\int}_0^T{wss}_i dt\right|}{\int_0^T\left|{wss}_i\right| dt}\right) $$

### Statistical analysis

Results were visualised using ParaView (Kitware Inc., Clifton Park, NY, USA) and Matplotlib and all data components were analysed using a Statistica v.13.3 package (StatSoft, Tulsa, OK, USA). The Shapiro-Wilk test was performed to assess the normal distribution of the investigated groups. Depending on the distribution, the parameters between groups were examined using the Student’s t-test or Mann–Whitney U test. To test if the proportion of cases characterised by a combination of high WSS and positive WSSG was significantly different between analysed groups, the Fisher’s exact test was used. *P*-values < 0.05 were considered statistically significant. For the sake of statistical analysis, dirWSS was encoded as follows: positive dirWSSG – 1, negative dirWSSG – 0. All haemodynamic parameters were subjected to logistic regression analysis with a stepwise addition mode. The potential risk factors for MCA aneurysm formation were identified on the univariate analysis. Intercorrelations between analysed parameters were examined using the Spearman’s rank correlation test. All variables were entered into a logistic regression model. Uncorrelated variables with *p*-values < 0.1 on univariate analysis were included in the multivariate logistic regression model to identify the independent predictors of MCA aneurysm formation. The results were presented as odds ratios (ORs) and their 95% confidence intervals (CIs). The independent predictors of an MCA aneurysm were subjected to receiver-operating characteristic (ROC) analysis to identify the area under the curve (AUC) values with optimal sensitivity and specificity.

## Results

### Haemodynamic parameters

As none of the haemodynamic parameters were normally distributed, they were analysed using the Mann–Whitney U test (Table 1). Patients from the case group had positive WSSG with significantly higher values (11.05 vs − 14.76 [Pa/mm], *P* = 0.020). The WSS, absWSSG and OSI values were not significantly different between the case and control groups (69.36 vs 76.59 [Pa], *P* = 0.158; 23.63 vs 35.07 [Pa/mm], *P* = 0.208; 0.175 vs 0.185, *P* = 0.759; respectively).

**Table 1:** Comparison of haemodynamic parameters in the analysed groups

Parameter	Case (*n* = 38)	Control (*n* = 39)	*P* value
WSS [Pa]	69.36 (59.27–89.96)	76.59 (61.92–125.27)	0.158
WSSG [Pa/mm]	11.05 (−14.88–28.06)	−14.76 (− 64.49–18.14)	0.020
absWSSG [Pa/mm]	23.63 (12.29–56.13)	35.07 (15.29–74.86)	0.208
OSI	0.175 (0.069–0.319)	0.185 (0–0.353)	0.759

*WSS* wall shear stress, *WSSG* wall shear stress gradient, *absWSSG* absolute WSSG, *OSI* oscillatory shear index; values are presented as median (IQR, 25th–75th percentile); analysed with the Mann–Whitney U test

**Fig. 2:**
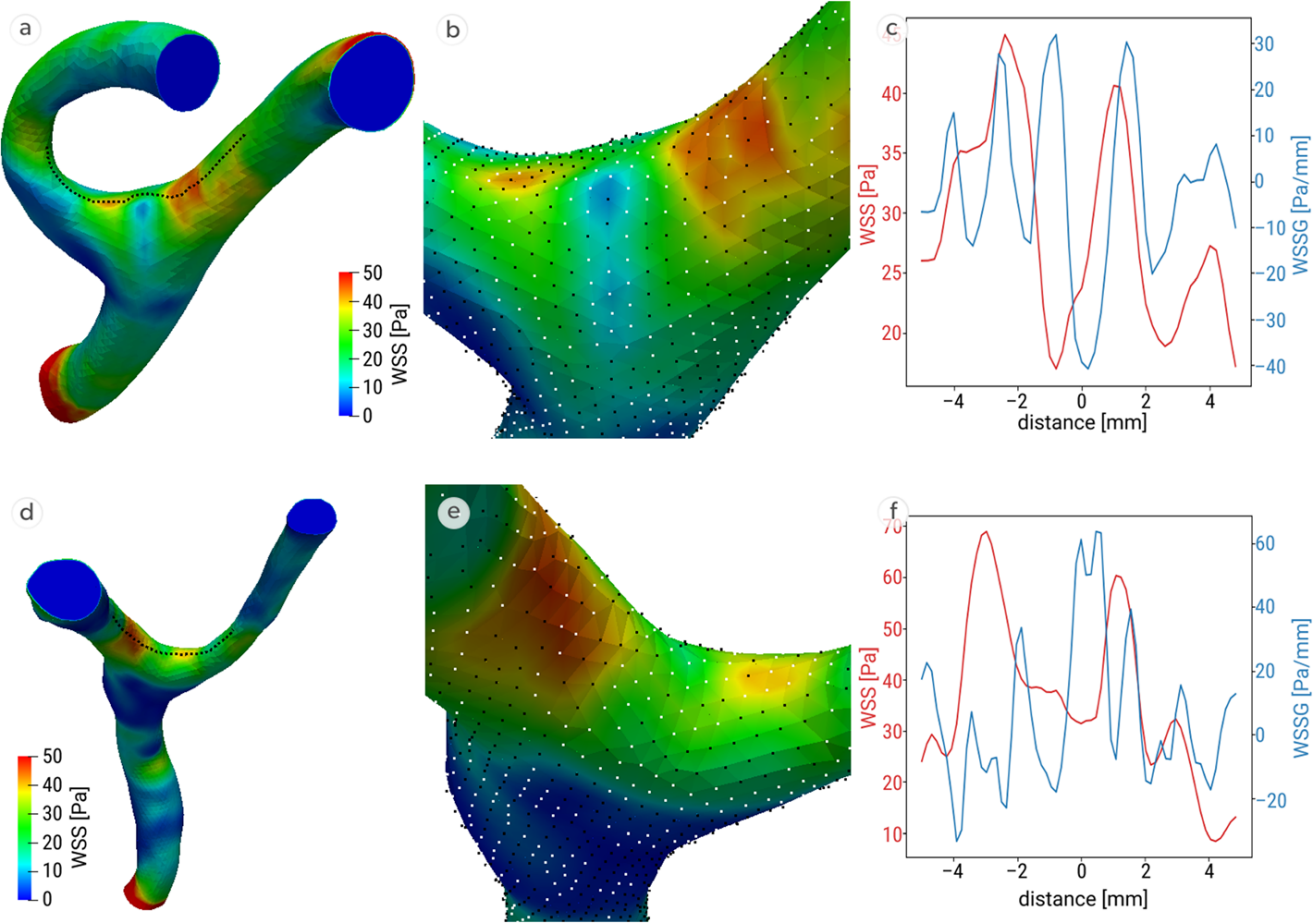
Distribution of WSS (**a** and **d**); bifurcation with points representing positive (white) and negative (black) WSSG (**b** and **e**); WSS (red) and WSSG (blue) distribution along the wall (**c** and **f**), WSSG and WSS measurements taken along the dotted lines (**a** and **d**); representative MCA bifurcation models from case (upper row) and control (lower row) groups. WSS – wall shear stress; WSSG – wall shear stress gradient

Fig. 2 shows the distribution of WSS and WSSG in exemplificatory MCA bifurcation models from both groups. Areas coloured red represent those with high WSS values while areas with low WSS values are blue. It should be noted that those places with the highest WSS were not located at the bifurcation apex but in close proximity. There were usually two places of relatively high WSS – one located on each branch (Fig. 2a, b, d, e).

Charts (Fig. 2c and f) present the characteristic haemodynamic patterns observed in both groups. The WSSG values near the bifurcation apex were positive and tended to decrease with distance, dropping below zero (Fig. 2c and f). Significantly more patients in the case group had haemodynamic regions of high WSS values combined with high and positive WSSG near the bifurcation apex (23/38), while in control group, high WSS values were usually accompanied by negative WSSG (14/39) (Table 2, *P* = 0.041).

**Table 2:** Comparison of regions of high WSS values combined with high and positive WSSG near the bifurcation apex in the analysed groups

Parameter	Case (*n* = 38)	Control (*n* = 39)	Total
dirWSSG+	23	14	37
dirWSSG-	15	25	40
total	38	39	P value = 0.041

dirWSSG+ − positive WSSG, dirWSSG- – negative WSSG; analysed with the Fisher’s exact test

In both groups, the regions of high WSS were located slightly more often on the larger branch (case group 55.3%, *n* = 38; control group 56.4%, *n* = 39). In the vast majority of cases (32/38), it was not possible to define from which branch the aneurysm had originated. In the remaining patients (6/38), the aneurysm locations corresponded with areas of high WSS and a positive WSSG, and overlapped the aneurysm ostia containing the initiation zone.

### Predictors of MCA aneurysm – logistic regression and ROC analysis

The univariate logistic regression analysis identified two parameters as significant predictors for MCA aneurysm formation: WSSG (OR: 1.008; 95% CI: 1.0–1.016; *P* = 0.028) and dirWSSG (OR: 2.738; 95% CI: 1.088–6.889; *P* = 0.030) (Table 3). Intercorrelations between the analysed parameters were examined using the Spearman’s rank correlation test. For obvious reasons, the WSSG values were significantly correlated with dirWSSG (correlation coefficient: 0.865, *P* < 0.0001). Additionally, absWSSG was correlated with WSS (correlation coefficient: 0.488, P < 0.0001) and with OSI (correlation coefficient: -0.309, *P* = 0.006). Other parameters were not correlated.

**Table 3:** Risk factors of MCA aneurysm formation: univariate and multivariate logistic regression analysis

Parameter	Univariate analysis		Multivariate analysis	
	OR (95% CI)	*P* value	OR (95% CI)	*P* value
WSS [Pa]	0.988 (0.975–1.002)	0.077	0.986 (0.971–1.0)	0.049
WSSG [Pa/mm]	1.008 (1.0–1.016)	0.028	1.009 (1.001–1.017)	0.025
absWSSG [Pa/mm]	0.994 (0.985–1.004)	0.207	–	–
dirWSSG	2.738 (1.088–6.889)	0.030	–	–
OSI	1.158 (0.071–18.84)	0.918	–	–

For abbreviations, see Table 1. Multivariate model included all uncorrelated variables with P value < 0.1 in the univariate analysis

For the final multivariate logistic regression model, we included two variables – WSS and WSSG – identified as factors significantly influencing MCA aneurysms formation (OR: 0.986; 95% CI: 0.971–1.0; *P* = 0.049; OR: 1.009; 95% CI: 1.001–1.017; *P* = 0.025, respectively) (Table 3). The logistic regression formula with the corresponding coefficients for WSS and WSSG is presented below:

$$ \frac{1}{1+{\mathrm{e}}^{-\left({\upbeta}_0+{\upbeta}_1\mathrm{WSS}+{\upbeta}_2\mathrm{WSSG}\right)}} $$

where β_0_ = 1.133, β_1_ = − 0.014, β_2_ = 0.009, WSS – wall shear stress, WSSG – wall shear stress gradient.

**Fig. 3:**
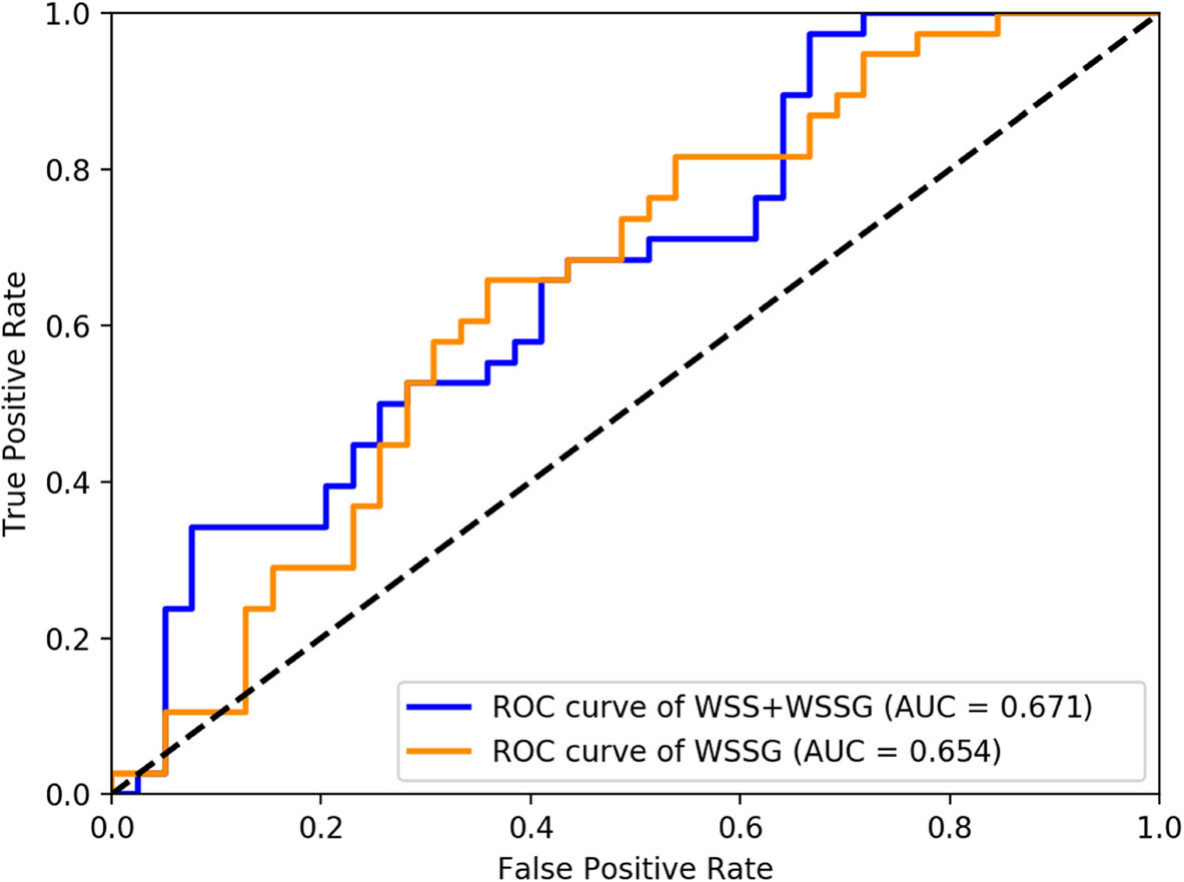
ROC curves for all of the most significant risk factors of MCA aneurysm formation. WSS – wall shear stress; WSSG – wall shear stress gradient

The ROC curve for discovered risk factors for MCA aneurysm formation is presented in Fig. 3. From the analysis of the ROC curve for WSSG, the area under the curve (AUC) was 0.654, with the optimal cut-off value −0.37 Pa/mm (sensitivity 0.658 and specificity 0.641). The largest AUC value was recognised for combined WSS and WSSG (AUC = 0.671).

## Discussion

The main findings of this study are: (1) cerebral aneurysm development might be an independent effect of haemodynamic factors and (2) aneurysms form near the bifurcation apices in regions of high WSS values accompanied by positive WSSG.

Haemodynamic forces are considered to provide a regulatory function for both the physiologic and pathologic aspects of endothelial activity in the blood vessels. Endothelial cells, through molecular sensors such as integrins and mechanosensitive ion channels located on their surface, transform the WSS-induced mechanical signals into biological signals, activating molecular pathways to maintain vascular homeostasis [21, 22, 28, 29]. Endothelial dysfunction is known to contribute to intracranial aneurysm pathogenesis [11]. Data reported from animal experimentation showed that damage or injury to the endothelium is usually the first incident in intracranial aneurysm formation [30]. Haemodynamic stress activates key molecular pathways that result in the upregulation of adhesion molecules and chemotactic cytokines leading to inflammatory zone formation and compromising the endothelial cell-to-cell junctions [22, 29, 31, 32]. These processes are based on the overexpression of a large group of genes, such as *ADAMTS1*, *PLAU*, *PLAT*, and *TIMP3*, all of which are involved in extracellular matrix (ECM) degradation, induced by very high WSS [17]. In vivo studies have shown that high flow and WSS induce significant proliferation of endothelial cells (ECs) and smooth muscle cells (SMCs), accompanied by significant increases in matrix metallopeptidases (MMPs) [18, 19] responsible for the degradation and digestion of extracellular matrix components. The high level of MMPs degrades cell basement membranes and internal elastic lamina (IEL), allowing proliferating ECs to migrate into the new enlarged areas and allowing proliferating SMCs to reorientate in the media [18, 19] leading, consequently, to aneurysmal wall remodelling. Many CFD-studies have demonstrated a positive correlation with high WSS in bifurcation aneurysms [33–37], whereas a negative correlation was found in sidewall aneurysms [12, 13, 38, 39]. Our study showed a similar increase in WSS at MCA bifurcation in both studied groups of patients (control and aneurysmal), which suggests that high WSS alone cannot initiate IA formation. Univariate logistic regression analysis performed in our study revealed only two significant predictors for MCA aneurysm formation – the WSSG value and its direction (dirWSSG), but only the WSSG turned out to be a significant independent prognosticator. Therefore, in our opinion, high WSS impacts on MCA aneurysm formation, whereas a positive WSSG mainly promotes this process. Additionally, the results of our study confirmed the lack of a correlation between OSI and bifurcation aneurysm formation, which is consistent with the previously published data [12, 40].

In in vivo studies on dogs in which arterial bifurcations were surgically created from native common carotid arteries with increased blood flow (animals were on a high salt diet, and some had one of their renal arteries ligated), combined with CFD simulations performed on the in vivo images and results mapped onto histological images, have suggested the coexistence of increased WSS and positive high WSSG at the bifurcation as triggers for aneurysm initiation [41, 42]. However, little is known about the effect of high WSS and the WSSG on the formation of IAs in humans. The majority of image-based CFD studies investigating quantitative haemodynamic variables and their relationships demonstrated a positive correlation between WSS and/or WSSG and bifurcation aneurysm formation [34–36], while studies performed for sidewall aneurysms denied such a correlation [12, 39, 40]. Of note, all the above-mentioned studies were mainly case series reports conducted on a small group of patients, whereas our clinical-control study was based on a large homogeneous MCA bifurcation group of 77 patients, which increased the statistical power of the analyses performed.

The complex influence of several haemodynamic factors, such as high WSS with corresponding positive WSSG values, may induce degenerative changes in the arterial wall and result in aneurysm formation [41–43]. According to the work by Dolan et al. [25], a positive WSSG induces gene expression that could promote the proliferation of ECs, apoptosis, and extracellular processing. Specifically, a positive WSSG downregulated *RPRM* and *BMP4* genes which are involved in cell cycle arrest and cell growth and differentiation, respectively [25]. In addition, a positive WSSG leads to downregulation of the genes that inhibit apoptosis (such as: *MCP1*, *CSF2*, *BMP4*, and *THBS1*) increasing the propensity of ECs to be apoptotic [25]. However, the key factor in the aneurysm formation process is the high WSS and positive WSSG induction of matrix remodelling. A positive WSSG increases significant ADAMTS1 expression in ECs (an ECM protease, which degrades ECM proteins including aggrecan, versican and thrombospondin), and downregulates TAGLN (a repressor of MMP-9 expression) further increasing ECM degradation [25]. All these processes make the arterial wall more vulnerable to mechanical damage [24, 25]. Interestingly, when taken together, a positive WSSG and WSS may augment the effect of high WSS on gene expression, as opposed to a negative WSSG that antagonises the effect of high WSS on gene expression [25].

Previous CFD studies were unable to identify strong risk factors or predictors for aneurysm formation among the known haemodynamic parameters (WSS, WSSG, OSI). This induced a strong need and motivation to find new haemodynamic parameters describing aneurysm initiation regions in a way previously unknown. Two new variables, derived from the previously described WSS and WSSG, were introduced specifically to study aneurysm formation: the aneurysm formation indicator (AFI) [13] and the gradient oscillatory number (GON) [12]. AFI quantifies the change of the WSS vector direction, by calculating the angle between the instantaneous and time-averaged WSS vector at any time point throughout the pulse cycle. Mantha et al. [13] found significant correlations between AFI and the location of sidewall aneurysm formation and concluded that the introduced parameter may be useful in identifying areas for future aneurysm formation. However, Shimogonya et al. [12] could not find significant correlations between previously analysed haemodynamic parameters (including AFI) and the location of aneurysm formation, so they proposed a new index for the initiation of a cerebral aneurysm – GON. GON quantifies fluctuations of the compression forces integrated over one cardiac cycle. Although, correlations between an elevated GON and the location of aneurysm formation were found [12], later studies [38, 40] showed that elevated GON values may be found in many non-aneurysmal sites, indicating that GON might be sensitive but not specific for aneurysm formation. The most recently proposed variable – transWSS (transverse wall shear stress) – which describes the multidirectional nature of disturbed flow, was strongly correlated with atherosclerotic lesions [44]. Due to its unique properties, Geers et al. [34] decided to assess transWSS influence in the context of aneurysm initiation. For both cases and controls, regions of high transWSS were concentrated, suggesting that flow disturbances remained in the same location throughout the cardiac cycle. Although they reported that cases were found to have significantly higher transWSS values, there was no clear correlation with the aneurysm initiation site.

Similarly to other authors, we found that the places of highest WSS were located not at the bifurcation apex, but in close proximity. Following flow impingement at a bifurcation apex, it splits into daughter branches and experiences rapid acceleration followed by deceleration creating areas of positive and negative WSSG. In the analysed bifurcations from the case group, we identified three haemodynamic regions previously discovered and described by Meng et al. [41, 42]: impingement, acceleration and recovery zones. As MCA aneurysms tend to form near the bifurcation apex, on one of the daughter branches, aneurysm ostia containing initiation zones were overlapping with regions of high WSS and positive WSSG.

The WSS experienced at a bifurcation is dependent on its geometry, including the radii of all the vessels involved and the bifurcation angle of the arterial tree [45, 46]. The increase in the asymmetry of the vascular diameters of bifurcations and the values of the bifurcation angles, and the decrease in the bilateral angles formed between the daughter and parent vessels and the inclination angle (formed between the parent vessel axis and the plane containing the axes of the two daughter vessels) likely contribute to abnormally enhanced haemodynamic stresses at the arterial bifurcations [26, 47–53]. Thus, arterial morphology and local haemodynamics are interrelated: the geometry directly determines the blood-flow parameters, while the flow drives arterial wall remodelling which determines the future geometry, promoting aneurysm formation.

## Conclusions

This study demonstrated that the development of cerebral aneurysms might be an independent effect of haemodynamic factors. Furthermore, we also demonstrated that high WSS impacts MCA aneurysm formation, while positive WSSG mainly promotes this process. There is a considerable need to study the role of the interactions between haemodynamic forces, arterial geometry and the arterial wall mechanobiology in the natural history of intracranial aneurysm formation in experimental models. Patient-specific fluid structure interaction simulations, involving interactions between the elastic arterial wall and flowing blood, must be enhanced by histopathology studies as these approaches are complementary and mutually informative. Analysis of such simulations may be helpful in discovering the exact mechanisms of IA formation and may enable us to develop new and more efficient diagnostic tools.

## Data Availability

The custom-written script used to generate patient-specific inlet velocity profiles is available online (see link: https://github.com/kudlacz964/tccs2ansys).

## Abbreviations

absWSSG: Absolute wall shear stress gradient
ACoA: Anterior communicating artery
AFI: Aneurysm formation index
AUC: Area under the curve
CFD: Computational fluid dynamics
CI: Confidence interval
CTA: Computed tomography angiography
dirWSSG: Direction wall shear stress
EC: Endothelial cell
ECM: Extracellular matrix
GON: Gradient oscillatory number
IA: Intracranial aneurysm
ICA: Internal carotid artery
IEL: Internal elastic lamina
MCA: Middle cerebral artery
MMP: Matrix metallopeptidase
OSI: Oscillatory shear index
OR: Odds ratio
PCA: Posterior cerebral artery
SAH: Subarachnoid haemorrhage
SMC: Smooth muscle cell
TCCS: Transcranial colour-coded sonography
Ved: End-diastolic velocity
Vm: Mean velocity
Vps: Peak systolic velocity
WSS: Wall shear stress
WSSG: Wall shear stress gradient
